# Surveying immigrants in Southern Europe: Spanish and Italian strategies in comparative perspective

**DOI:** 10.1186/s40878-017-0060-4

**Published:** 2017-11-03

**Authors:** Inmaculada Serrano Sanguilinda, Elisa Barbiano di Belgiojoso, Amparo González Ferrer, Stefania Maria Lorenza Rimoldi, Gian Carlo Blangiardo

**Affiliations:** 1 National Research Council, CSIC, Albasanz, 26-28, 28037 Madrid, Spain; 2 Università degli Studi di Milano-Bicocca, Via degli Arcimboldi 8, 20126 Milan, Italy

**Keywords:** Sampling migrants, Population register, Italy, Spain

## Abstract

Italy and Spain, as countries of recent immigration and high irregularity rates, have struggled to adapt their statistical system, especially their population registers, to adequately reflect the presence of an increasing number of immigrants in their territory. The population registers of the two countries have adapted differently to these changing realities: Spain introduced significant improvements in Padrón which have increased its coverage and accuracy. This is still not the case in Italy, making it necessary to resort to non-random sampling methods. The paper discusses the methodological implications of these differences and evaluates different methodological solutions based on both random and non-random sampling methods in both countries.

## Introduction

Achieving a representative sample of immigrant populations faces two fundamental problems: the availability of sample frames that correctly identify these populations and elevated fieldwork costs due to territorial dispersion, among other factors. Population registers provide the most updated and comprehensive sample frame on a country’s population, but they do not always contain the necessary categories to identify immigrant population, or their coverage of this target population is imperfect, which also hinders the representativeness and unbiased nature of samples obtained from these registers. This article focuses on the comparison of two similar migration contexts, Spain and Italy, and the specific challenges these countries encounter as recent destination countries with high levels of irregularity among their immigrant population. The population registers in both countries, *Padrón* and *Anagrafe*, respectively, have adapted differently to these changing realities. The paper discusses these differences and analyzes the methodological implications for sampling immigrant population in both countries and in comparative perspective.

The paper proceeds as follows: Short description of the immigration to Italy and Spain briefly describes recent immigration to both Italy and Spain. *Padrón* and *Anagrafe*: statistical categories available to identify immigrant population discusses the statistical categories needed for identifying migrant population in the contexts of recent migration, and evaluates the *Padrón* and *Anagrafe* along these lines. Padrón and Anagrafe: coverage, accuracy and access discusses additional issues of coverage, quality and access for the *Padrón* and *Anagrafe*. Implications for register-based probability sampling of immigrants in Spain and Italy analyzes the challenges and limitations of register-based sampling in both countries, assessing the outcomes of some empirical experiences and Alternative sampling methods for immigrants in Spain and Italy evaluates the experience of different alternative sampling methods. Discussion and conclusions concludes with a discussion on the implications of the Anagrafe and Padrón characteristics and an evaluation of the different methodological solutions presented.

## Short description of the immigration to Italy and Spain

Italy and Spain, which had long been emigration countries, have converted into immigrant nations, with an increasing number of immigrants coming from less developed countries (Bonifazi, 2013; Strozza, 2004; Arango, 2000). In Spain, the foreign-born population, which represented less than 2.5% of the total population at the end of the 90s, increased to more than 6 million people (15% of total) in 2016, according to the data from the Population Register (National Statistical Institute, INE). That same year, the foreign population legally residing in Italy amounted to more than 5 million people (approximately 8% of total population), compared to a total of less than 3% at the beginning of the century (Population Register, Italian National Institute for Statistics, Istat).^1^ If irregular (non-EU) migrants (435,000) and regular but unregistered migrants (410,000 – mainly from EU countries) are added, the total foreign population in Italy reaches approximately 6 million people, according to the latest estimates by Blangiardo (2016).^2^

Among non-EU residents, a high incidence of irregularity has been traditionally identified as a defining characteristic of the immigration regime in both countries. This is largely the result of relatively loose visa policies and large informal sectors in the labour market (Arango & Finotelli, 2009; Cebolla & González-Ferrer, 2008; Reyneri, 1998; Strozza, 2004). However, successive regularization programs^3^ have substantially reduced this irregularity rates: in Spain, from 60% in 2003 to 13% in 2008 (Cebolla & González-Ferrer, 2013), and in Italy, from 62% in 1990 to 7% in 2016 (Blangiardo, 2016). Additionally, the proportion of non-EU foreigners with a permanent residence permit increased in Spain from 36% in 2008 to 68% in 2012, which clearly illustrates the settlement process of the immigrant population in Spain (Cebolla & González-Ferrer, 2013). In Italy, 45.7% of the foreign resident population in 2015 had arrived before 2003, meaning they have been living in the country for more than 10 years, and 57% have a permanent residence permit (Istat, 2015).

The economic crisis significantly changed the migration dynamics. In Spain, emigration progressively increased for both immigrants and natives, especially since 2011 (Domingo & Blanes, 2015; González-Ferrer, 2013), and immigration has substantially declined since 2008, although annual entries remain still at high levels (more than 250,000 in 2013). In Italy, net migration started to fall in 2008 (450,000) until in 2014 it reaches the same level registered at the turn of the century (143,000 units) (Blangiardo, 2015).

## *Padrón* and *Anagrafe*: statistical categories available to identify immigrant population

Immigrant populations living in a particular country at a certain moment can be identified using two main criteria: country of birth (foreign-born vs natives) or citizenship (non-citizens vs citizens). Country of birth offers the advantage of being a stable, time-constant individual characteristic, whereas citizenship can be multiple and it can change over time, which may disguise migrant status. However, statistics based on citizenship allow to better distinguish between foreign migration inflows and returns of national citizens who migrated abroad (Strozza, Natale, Todisco, & Ballacci, 2002). But neither of these categories can fully identify the immigrant population in a country (Jacobs, et al., 2009; Méndez & Font, 2013), since the two categories do not perfectly overlap, and both are relevant to identify a segment of the population with peculiar legal and social links to the host country.

In Italy, official statistics are generally organized according to the concept of citizenship.^4^ Non-EU migration inflows are counted through permits of stay, which are obviously granted only to foreign citizens. Inflows of EU citizens, as well as stocks of the overall population, are counted through the population register (*Anagrafe*).^5^ In Spain, citizenship was also traditionally the main concept organizing data collection in the field of migration but, as immigration flows increased, information on country of birth has become increasingly available. The Municipal Register (*Padrón*) first included information on country of birth in 1996, although this information was not exploited in the data periodically published by the Statistics Office (INE) until years later (see more in González-Ferrer, 2009). The Statistics on Residence Permits did not include this information until 2005. Currently, the INE systematically includes this variable in most of its statistical operations, together with the variable of citizenship.

Opting for the citizenship criterion to organize immigration figures seems reasonable in Italy considering the quite restrictive laws on the acquisition of the Italian citizenship (10 years of residence, marriage with an Italian and 2 years of residence, and by formal request after turning 18 years old for foreigners born in Italy) and the long emigration experience of Italians. However, as the size and length of settlement of international immigrants in Italy increase, acquisitions of Italian citizenship have also increased from about 50,000 in 2008 to 200,000 in 2016 (Blangiardo, 2015; Istat, Demographic Statistics). Moreover, by now the return migration of Italian emigrants is almost negligible.

The systematic incorporation of the country of birth variable in Spanish statistics must be particularly welcome considering the rules regulating the access to Spanish citizenship. Even if the general rule is the same as in Italy (10 years of residence) Latin Americans, which represent a large proportion of the foreign-born population in Spain (56% of the non-EU citizens) are allowed to apply for Spanish citizenship after 2 years of continuous legal residence in Spain. A total of 1.270 million persons have in fact obtained the Spanish nationality between 1996 and 2015 (Ministerio de Empleo y Seguridad Social). In this context, having statistical information on immigrants only on the basis of current citizenship would represent a major limitation.

## Padrón and Anagrafe: coverage, accuracy and access

### Coverage

Italy and Spain are characterized by high levels of irregularity among their immigrant population, even if those levels have descended in recent years, making it a particularly salient issue. In Italy, nonetheless – as it happens in most countries around the world – irregular migrants cannot get registered in *Anagrafe*. In contrast, since 1996 Spanish municipalities not only have the possibility to register every person living in their territory regardless of their administrative status, but also the obligation to do so.

Coverage is also affected by the incentives and barriers to registration. In Italy, the registration in the *Anagrafe* is mandatory for both non-EU foreign citizens (with residence permit) and EU citizens (after 3 months of presence), but this is not an automatic process. The registration, in fact, requires a voluntary inscription that bears little or no incentives, which leads to significant under-registration – amounting to 410,000 individuals in 2015, particularly among EU migrants (Blangiardo, 2016). In contrast, the registration in the Spanish *Padrón* counts with very strong incentives since the year 2000, when the Law 4/2000 on the Rights of Foreigners and their Social Integration into Spanish Society imposed the *empadronamiento* (registration in the Padrón) as the only legal requirement to access public health care and other public services such as enrollment in primary schools. In addition to this, the regularisation program that accompanied the legal reform – and all other regularisations implemented since – accepted the certificate of *empadronamiento* as a proof of the length of residence in Spain, which especially encouraged the registration of undocumented migrants. Last but not least, most municipalities carried out campaigns to promote the *empadronamiento* of foreign residents in those early years, since the budget they receive from the central government depends precisely on their total population as counted in the *Padrón*.

### Accuracy

The accuracy and reliability of population registers also depends on the timely de-registration of persons when they leave the country – as well as notification of changes in their residence within it. This general problem is particularly relevant in the case of migrants, who are a more mobile population (Reichel & Morales, 2017). In both Italy and Spain de-registration is not mandatory, and there are no particular incentives for migrants to deregister when they leave permanently. This situation results in over-registration, reducing the reliability of these registers to identify (and sample) immigrant-origin population.

In Italy, cleaning operations of the *Anagrafe* are conducted only after every Census, when registrations and (above all) de-registrations ‘d’ufficio’ are possible. Spain additionally introduced in 2005 an ‘expiry procedure’ (*caducidad padronal*) implemented for the first time in 2007, that requires all non-EU foreigners without a permanent residence permit to renew their registration in the Padrón every 2 years. After its first implementation, the new population figures for January 1st, 2007 reflected that approximately a quarter million non-EU foreigners had been deregistered ‘ex officio’ (González-Ferrer, 2009).^6^ Secondly, a periodical residence check (*comprobación periódica de la residencia*) to be conducted every 5 years was introduced for EU citizens and permanent non-EU residents in 2011. This procedure was implemented for the first time in 2013: more than 400,000 foreigners disappeared from the *Padrón*, 87% of which were EU citizens (mainly from Germany, UK and France) (Ródenas, 2012).

### Access

In Spain, it is feasible to draw individual probability samples for both foreigners and foreign born individuals living in the country from the *Padrón*. Individual samples from the *Padrón* can be requested from the National Statistical Institute (INE). However, official support from some unit of the Spanish Public Administration is required and public universities, even Spanish ones, do not qualify as such. Public universities can benefit though from a 15% discount in the final price for the sample. Access to the *Anagrafe* for research is strictly limited, since only municipalities are entitled to manage it: anonymous records for research sometimes can be obtained at the discretion of the public officers. Even the Italian Institute of Statistics (Istat) does not have access to the individual records, and the selection of the sample in the case of surveys is carried out by the municipalities.

## Implications for register-based probability sampling of immigrants in Spain and Italy

### Italy: the limitations of the Anagrafe to sample immigrants

In Italy, the coverage of *Anagrafe* is limited to the subgroup of foreigners (but not foreign-born) who have voluntarily registered in the *Anagrafe* and, in the case of non-EU citizens this excludes migrants without a residence permit*.* This limited coverage has led various authors (Natale & Strozza, 1997; Strozza, 2004; Blangiardo, 1996) to discard register-based probabilistic sampling.

However, Istat still uses this approach and has conducted several surveys on households including at least one foreign member (see Table 1). These surveys are of crucial importance to deepen our knowledge of the foreign population living in Italy and to fill statistical gaps between censuses. However, it is necessary to bear in mind its significant limitations.

**Table 1:** Ad hoc surveys on foreign population conducted by ISTAT using the population register (Anagrafe)

Survey	Themes	Sample size	Response rate	Strategies for improving the quality of samples and surveys	Advantages/Limits or problems	Available documentation
Health and use of health services (2005)	Health problems, use of health services	3509 foreigners	N.A.	–	First survey on the topic No territorial stratification; Questionnaire in Italian only	http://www.Istat.it/it/immigrati/prodotti-editoriali/salute-e-sanit%C3%A0 (Language: Italian)
The integration of migrants and their descendants in the labour market (2008) - Ad hoc module in the Labour Force Survey	Insertion into the Italian labour market	1896 foreigners and naturalized	N.A.	–	Combined use of citizenship and country of birth, both of the interviewees and their parents	http://www.Istat.it/it/archivio/8581 (Language: Italian)
Income and living conditions among household with foreigners (2009)	Economic condition of the foreign household	6000 households and 15,000 individuals	66,3%	Questionnaire translated in 10 foreign languages	Comparison with the Italian population	http://www.Istat.it/it/archivio/52405 (Language: Italian)
Social condition and integration of foreign citizens (2011–2012)	Household, education, migration process, working trajectory, discrimination, health and integration	9500 households and 25,000 individuals	89% (refusal rate 8%)	Substitute sample of households (similar with respect to citizenship, geographic residence and household size)		http://www.Istat.it/it/archivio/10825 (Language: Italian)
The integration of foreigners and naturalized citizens in the labour market (2014) - Ad hoc module in the Labour Force Survey	Insertion into the Italian labour market, motives for migration and difficulties in the labour market	N.A.	N.A.	–		http://www90.Istat.it/en/archive/177536 (Language: English) http://ec.europa.eu/eurostat/documents/1978984/6037334/Evaluation_report_AHM_2014.pdf (Language: English)
Labour Force Sample among foreigners (as a part of LFS, continuously)	labour participation of people aged 15 and over	As a part of the overall sample	N.A.	Substitute sample of households (similar with respect to citizenship, geographic residence and household size)	Comparison with the overall population and with other countries	

Most importantly, sampling from the population register leads to selecting only regular and resident migrants, which are the most stable segment of the population of interest (e.g. migrants with a longer length of stay and more frequently reunited with their relatives). The resulting samples will be less useful in studying the less stable population that goes unregistered in the *Anagrafe* (recent arrivals, irregular situation or short-term migrants).^7^

Additionally, the coverage and accuracy of the register are also imperfect for the more stable population, due to the voluntary nature of registration and de-registration. Inaccuracy in the register also leads to increased non-contact rates. For instance, in the “Social condition and integration of foreign citizens” surveys, the final sample of households ended up being substantially smaller than initially expected in the sampling design (9553 compared to 12,269). This was so despite the fact that, in order to guarantee a sufficient sample size, the number of selected households was three times the theoretical sample size: each sample unit had three possible alternatives (similar with respect to citizenship, geographic residence and household size) to be used in case of the impossibility of contacting the first household (Istat, 2016).

### Spain: the challenges of sampling immigrants from the Padrón

In Spain, the coverage of *Padrón* is more comprehensive, including both foreign-born and foreigners, and both regular and irregular (non-EU) migrants. Furthermore, there are strong incentives to register in the *Padrón* for all members of this population, including irregular migrants. But even if an adequate sample frame is available, the problem of elevated costs of fieldwork remains – due to the relatively small-size of migrant populations scattered across the territory, relative mobility and vulnerability (Reichel & Morales, 2017). This difficulty is compounded when the sample frame is inaccurate due to a lack of de-registration or notifications of change of residence, which affects contact rates (and might potentially introduce biases in the final sample). De-registration is voluntary and no particular incentives to do it exist in Spain either. However, the two corrective procedures – the 'expiry procedure' for non-EU non-permanent foreign residents and the ‘periodical residence check’ for EU citizens-, which fully deployed their effects in 2006/7 and 2013 respectively, should have increased the reliability of the registry and lead to relatively improved contact rates.

The INE was the first institution to use the Padrón to carry out an individual probability sample to survey immigrants in Spain (National Immigrant Survey, ENI 2007). More focused immigrant surveys have been conducted as well by specialized research teams with less resources and more specific goals, often focused on particular origin groups and/or limited geographical areas. The most relevant ones are summarized in Table 2.

**Table 2:** Surveys conducted with immigrant origin populations in Spain using random sampling based on the population register

Survey	Topics & duration questionnaire	Geographic coverage	Target population & sample size	Refusal rate	Strategies for improving the quality of samples and surveys	Advantages/Limits or problems	Available documentation
ENI 2007	characteristics of migrants, their family relationships and their social networks	National	Foreign-born population (15,500)	Response rate 87,4%	sampling unit was the dwelling and replacement of dwellings from a substitute sample	Representative sample (national and per main groups)	http://www.ine.es/daco/daco42/inmigrantes/informe/eni07_informe.pdf
Localmultidem Survey 2006	Political behaviour and attitudes (50 min.)	Cities of Barcelona, Madrid, and Murcia	Per city: Morocco (300), Ecuador (300), Andeans (300) Spanish born (300)	N.A.	sampling units were dwellings and adjacent dwellings	Random sampling needed to be complemented with spatial and route sampling to achieve target sample size	Morales & Ros (2013)
MAFE 2008	Family, labour, legal & migratory trajectories (70 min.)	12 provinces	N: 200		Selection of provinces with highest concentration of Senegalese population		Beauchemin & González-Ferrer (2011), Beauchemin (2012)
MESE 2012	Family, labour, legal & migratory trajectories (83 min.)	12 provinces	N: 400	8.8%	Selection of provinces with highest concentration of Senegalese population replacement of individuals from a substitute sample and increased number of trips		N.A. See discussion below
ICS 2012	Integration (45 min.)	Cities of Barcelona & Madrid	Barcelona, N: 411 Madrid, N: 583	6.1 and 8.3%	sample was clustered by neighbourhood		Morales, Martínez, & Méndez (2012) Reichel & Morales (2017)
ISSP 2012	Health Care (29 min)	Spain	N: 4000 Foreigners: 482	2.3%	–	Comparison with general population Only foreigners	CIS (2012)
ISSP 2014	Citizenship (29 min.)	Spain	N: 3000 Foreigners: N.A.	4.4%	–	Comparison with general population Only foreigners	CIS (2014)

The first pilot for ENI was carried out in 2006, before the effects of the ‘expiry procedure’ were fully reflected in Padrón files from which the samples are drawn. The ENI pilot in fact revealed an elevated rate of non-contacts (36.2%), mainly because the selected person had moved to another address (19.4%). INE then decided to implement an alternative strategy in which the sampling unit was not the foreign-born individual but dwellings with at least one foreign-born occupant, also allowing the replacement of dwellings from a substitute sample (Duque, Ballano, & Perez, 2013).^8^ Almost at the same time, the Localmutidem survey was also conducted, aiming to sample 900 migrants from Morocco and the Andean region. After encountering difficulties to locate individuals, with non-contact rates reaching 59% (depending on the origin group), the Localmultidem survey also opted for expanding the sampling units from individuals to dwellings and adjacent dwellings.^9^

All other surveys in Table 2 were carried out once the first cleaning of the Padrón resulting from the ‘expiry procedure’ for non-EU migrants was fully implemented^10^, and all of them achieved their final numerical targets by applying an exclusively individual random sampling based on the *Padrón*. Still, non-contact rates remained elevated and different strategies were applied in order to maintain the survey costs within reasonable limits due also to territorial dispersion. For instance, in the case of ICS 2012 – aiming to sample at least 400 migrants in Madrid and Barcelona, respectively – the sample was clustered by neighbourhood code and selection was proportional to the size of the target population in the cluster, in order to reduce the time and money implied by an individual sample very dispersed throughout the territory. The results are deemed representative of the target population aside from random sampling errors or any biases that might be introduced by non-response (Reichel & Morales, 2017; Morales et al., 2012).

The MESE survey^11^ – aiming to sample 400 Senegalese-born immigrants – faced particular challenges due to the demanding nature of its biographical questionnaire, which took on average 83 min to complete^12^ and the peculiarity of its target population. Senegalese migrants are one of the most recent and vulnerable immigrant groups in Spain: they have a high irregularity rate (31,6% in 2008), they intensely concentrate in precarious jobs (Obućina, 2013) – which makes them more susceptible to internal mobility – and they are more residentially segregated than other groups (Díaz Hernández, Domínguez Mujica, & Parreño Castellano, 2010). All these factors are expected to produce high non-contact and refusal rates. The MESE sample was also designed to be directly proportional to the actual distribution of the Senegalese born population in the selected provinces, and as a result of this, it included a non-negligible number of municipalities with only one or two contacts, largely in rural areas. These highly dispersed contacts seemingly raised the cost of fieldwork.^13^ However, finding individuals in rural areas and obtaining a positive response to participate was actually much easier than in urban areas, which needs to be taken into account when assessing the pros and cons of geographical dispersion of immigrant samples.

In order to deal with the double challenge of high non-contact rates (particularly as a result of the high mobility of the population, moving within the territory following job opportunities and visiting Senegal as well) and dispersion of the sample, the MESE team introduced more flexibility in contacts replacement. When visiting a particular location within their assigned area, and if unable to find the individuals initially sampled, the interviewers were allowed to try visiting those replacements who lived nearby, as far as gender and age group was the same. Additionally, the number of geographical trips allowed for each interviewer was increased, so that they could exhaust the number of visits per contact and the contact list provided by *Padrón*.

MESE required, on average, 2.8 contacts from the *Padrón* sample to complete one interview, compared to 2.7 in ICS (Morales et al., 2012), 2.3 in ISSP 2012 (CIS, 2012), and 3.4 in ISSP 2014 (CIS, 2014); therefore, quite in line with previous studies that addressed the overall immigrant population, and not just one of most vulnerable groups within it. The same happened with regard to the non-contact^14^ and refusal rates (see Table 2) in spite of a much longer and complex questionnaire in MESE and, in principle, a more difficult target population.^15^

## Alternative sampling methods for immigrants in Spain and Italy

The use of population lists for sampling is a gold standard for obtaining representative probabilistic samples (Reichel & Morales, 2017). However, when population lists are not available or cannot adequately identify the target population, alternative sampling methods can be used.^16^ This is the case of Italy, where the lack of an exhaustive list of the target population (and the inaccessibility of the *Anagrafe*) led researchers since the 1980s to design and employ sampling strategies specific to surveying migrants (Natale & Strozza, 1997; Strozza, 2004; Blangiardo, 1996; Birindelli & Blangiardo, 2010). In Spain, where the *Padrón* constitutes a proper sample frame, researchers have kept turning to alternative (cheaper) sampling methods due to the financial and administrative barriers to obtain individual samples, and to the high cost and uncertainty associated with register-based random sampling.

The application of these methods faces similar challenges in these very similar contexts: ensuring the representativeness and adequacy of the sample, and again keeping the costs of sampling under control. Below we discuss the different methodologies and outcomes of different empirical experiences conducted in Italy and Spain, paying particular attention to the “Centre sampling technique” (CS), which has proved to be a very effective approach for addressing the Italian situation with many immigrants not registered in the *Anagrafe*.

### Other alternative sampling methods applied in Italy and Spain: outcomes and assessment

Table 3 describes the main methodologies applied in Italy and Spain, and their corresponding empirical experiences. The limitations of these methodologies and experiences are discussed below.

**Table 3:** Alternative sampling methods in Italy and Spain

Methodology	Sampling design	Advantages	Problems and difficulties	application
Snowball	K stages	Useful for pilot survey	Choice of initial list Attrition rates The probability of inclusion is unknown Full network connectivity assumption	ITA Interuniversity survey on “Foreigner Immigration to Italy”
Respondent driven sampling	Each municipality was divided into four areas, and one eligible immigrant was selected in each of them. First respondents in each area were not allowed to know each other to avoid overlapping networks; and each respondent could provide up to three contacts for other immigrants who had never lived in the same household as the respondent. The procedure was repeated until the final number of questionnaires was achieved.		over-representation of men, especially in the age group 30-39	SPA Romanian Communities in Spain, 2008. Four municipalities in the region of Madrid. (Sandu, 2009)
Residential units method (unità abitative)	2 stages List of apartments with at least a foreigner (1° level unit); random selection of one foreigner (2° level units). The initial list is updated through snowball question.	Lower non-response rate	Not suitable in big cities High attrition rate (snowball component)	ITA Casacchia & Strozza (1990) Natale & Strozza (1997)
Quota sampling	Quotas by age and gender in 4 Italian Regions. Selection channels: from contacts obtained in Senegal, or public spaces, migrant associations, snowballing, interviewers’ contacts		Multiple steps with high sample attrition and (non-random) selection selection biases at origin	ITA & SPA (partial sample) MAFE 2008. Beauchemin & González-Ferrer (2011)
Random routes	3 regions with largest percentages of the targeted population Districts where more than 10% of the population belonged to the target population To select the final respondents, random routes were combined with a spatial sampling method (see below)^a^	age distribution and the average length of stay of the surveyed population was quite similar to the Padrón distribution	women were seriously under-represented in most origin groups	SPA Remittances of Latin-American immigrants in Spain 2012. Adult immigrants from six Latin American countries (Izaguirre Vizcaya, et al., 2016).
Spatial sampling (and random routes)	Sampling in centres of aggregation: immigration information offices, public spaces in neighbourhoods and, above all, the queue outside the Moroccan consulate	refusal rates were low – especially among the individuals who were queuing for long hours outside the consulate premises	often the inability to locate anyone in certain public spaces required changing the intersection points	SPA Localmultidem 2006^b^ (Morales & Ros, 2013).
Centre sampling technique	2 stages List of centre of aggregation (1° level unit); casual selection of one foreign (2° level units).	Non response rate due only to refusal Suitable also to big cities Cost-efficient method Sample more representative and with weighted probabilities	Target population “present population” Use a shorter questionnaire	ITA Regional surveys: Lombardy Regional survey every year since 2001 National surveys: South survey aimed at studying effects of the 2003 regularization (Blangiardo & Farina, 2006) Integrometro survey aimed at gathering information on the integration process (Cesareo & Blangiardo, 2009). PER.La survey, to analyze the working trajectories of the migrant population (Ismu, Censis and Iprs, 2010). International surveys: Italian sample in the “Push and Pull factors” (Birindelli, et al., 2000) in the Italian, Portuguese, and Hungarian samples of the “Immigrant Citizens Survey” (Huddleston & Tjaden, 2012); in the sample of Milan for the Localmultidem survey (Morales & Pilati, 2014).

^a^1) routes were designed in a way that included concentration points for the target population (surrounding areas of supermarkets, malls, banks, bus and metro stops, health care centers, call shops, remittances agencies, bars, restaurants, churches, pharmacies, parks, etc.); 2) interviewers chose respondents from the resident population from passersby on the “random” walk. Two rules had to be respected: a) no more than 10 contacts or interviews could be completed in the same point on the same day and, b) only one member of the same family could be interviewed (in case more than one eligible respondent was identified)

^b^Due mostly to deficiencies in the sampling frame (Padrón prior to expiry procedure) and the impossibility of obtaining a new random sample from the register to complete the study on time, 267 interviews out of the 600 to complete the Moroccan subsample in the Localmultidem Survey in Madrid and Barcelona had to be obtained through spatial sampling

The application of the snowball method, one of the most traditional non-random methodologies, presents some difficulties in determining the initial list and thus risks introducing significant biases, and the probability of inclusion in the sample is unknown. Moreover, the assumption of full network connectivity implies that if members of a rare population do not know each other they will be excluded by the sample (Reichel & Morales, 2017; Natale & Strozza, 1997). Attrition rates can also be high (increasing fieldwork costs) since the methodology relies on obtaining contacts for other migrants and being able to contact them later (McKenzie & Mistiaen, 2009). The respondent driven sampling, similarly to snowball, implies relevant bias risks (Beauchemin & González-Ferrer, 2011). In the experience of the *Romanian Communities* (Sandu, 2009) the sample over-represented men, especially in the age group 30–39. The residential units method was introduced by Casacchia and Strozza (1990) at the beginning of the 1990s in Italy and its application was successful in small and medium cities of some Italian Region, but it failed in big cities due to the difficulties to locate the apartments and contacts indicated with the snowball question (Natale & Strozza, 1997). Quota sampling based on snow-balling was used in the MAFE survey (2008) in Italy – and partially in Spain. The authors find significant selection biases at origin (by age and socio-economic status) and, interestingly, much higher different response rates in Italy than in Spain.^17^ The authors reject the hypotheses of different implementing agencies or higher rates of irregularity in Italy, and propose that the particular political context at the time in Italy increased mistrust among respondents. Random routes have also been implemented to survey migrant population in Spain, but have only been successful used in combination with spatial sampling, the methodology most similar to the CS – see discussion below in the Localmultidem project. Spatial sampling produced low refusal rates and, overall, the socio-demographic profile of the final sample was quite similar to that of the ENI 2007. Substantive analyses also found no significant effect of the sampling method on a number of different variables related to the socio-political attitudes and behaviours of the respondents (Morales, Anduiza, Rodríguez & San Martín, 2008).

### Advantages and limitations of the “Centre sampling technique” (CS) for sampling immigrants in Italy

In the early 1990s, Blangiardo (1996) introduced the “Centre sampling technique” (CS). The CS is a probabilistic multi-level sample method specifically designed to collect information on a representative sample of migrants when a (complete) list of the target population is not available, and the total number of the population is unknown (Baio, Blangiardo, & Blangiardo, 2011). The rationale of the CS is that migrants, during their everyday life, frequent a range of ‘aggregation centres’ (such as immigrant specific services, phone centres, churches, etc.), which can be used as first level unit of analysis to select migrants (the second level unit). The CS methodology provides a scheme to estimate a set of weights to correct the initial probability of inclusion in the sample (Fasani, 2008; Kraler & Vogel, 2008; Vogel & Kovacheva, 2008).

Based on the evaluation of these experiences, different experts consider the estimates based on the CS to be the most accurate and reliable for Italy (Fasani, 2008; Mecatti & Migliorati, 2003; Blangiardo, 2008). In particular, the CS method provides reliable estimates of the percentage of registered foreigners in *Anagrafe* at the time of the survey (i.e. the *rate of residents* per 100 presents), and also the rate of foreigners with regular status with respect to residence (i.e. the *rate of regulars* per 100 presents).^18^ Furthermore, the estimations of irregular migrants produced after the 2003 regularization were successfully compared with the number of applicants for amnesty in 2006 (Barbiano di Belgiojoso & Rimoldi, 2007; Accetturo & Infante, 2013).

The main limitation of the CS is related to the composition of the target population by nationality: communities which are more closed and with lower levels of social participation are more difficult to detect and smaller communities have lower probabilities of being captured by the list of centres identified. For correcting this issue, the identification of the list of centres is a crucial point. It is necessary to identify the centres with a preliminary analysis of the environmental and socio-economic context, so that the set of centres is adequately heterogeneous and so that every subject in the universe is, at least theoretically, reachable at one of the selected centres.^19^ The CS strategy seems to guarantee better results when the origin mix in the immigrant population remains relatively stable over time.^20^

In terms of keeping the sampling costs low, a good level of cooperation among the selected respondents has also been found, compared with other surveys (Méndez & Font, 2013), and the non- response rate is about 30% on average.

## Discussion and conclusions

The use of population registers for sampling is a gold standard for obtaining representative probabilistic samples (Reichel & Morales, 2017), however they do not always contain the necessary categories to identify immigrant population, or their coverage of this target population is imperfect, which also hinders the representativeness and unbiased nature of samples obtained from these registers. This article focuses on the specific challenges to sample immigrant population in two recent destination countries, Spain and Italy, which furthermore register high levels of irregularity.

The *Padrón* in Spain seems to have adapted much better to the new migratory circumstances of the country, by incorporating the category of country of birth, enabling the registration of irregular migrants and providing strong incentives for the registration of the migrant population in general, and irregular migrants in particular. This is not the case of *Anagrafe*, where the available statistical categories are more limited (only citizenship, but not country of birth), undocumented migrants are excluded from the register, and there is a lack of incentives for registration. The implications of these limitations are significant for the correct identification and characterization of the migrant population in Italy, as well as for conducting comparative analyses with the Spanish or other similar cases.

First, the availability of both country of birth and citizenship criteria to identify the immigrant population is particularly desirable in contexts where the frontier between immigrants and natives, and between foreigners and nationals become blurred due to increasing numbers of naturalised residents, a growing second generation population and the return of past and recent emigrants, which is the case for both Italy and Spain (Blangiardo, 2016; Martín-Pérez & Moreno-Fuentes, 2012; González-Ferrer & Trilla, 2015).

Second, irregular migrants are a particularly vulnerable segment of the migrant population, of crucial importance for the integration process and the political debate; and at the same time particularly hard-to-reach and invisible for the administration. For these reasons, the identification and analysis of irregular migrants is one of the most challenging aspects of migration studies, and their exclusion from the *Anagrafe* is a significant limitation, particularly in a context of with relatively high irregularity rates. In contrast, the Spanish *Padrón* is quite a exceptional source worldwide concerning the statistical coverage of foreign population in an irregular administrative situation, which allows the inclusion of this vulnerable segment of the population in probabilistic register-based samples.

Finally, the lack of incentives for registration in Anagrafe also leads to a significant under-registration (and thus under-estimation) of EU and regular non-EU migrants. The overall gap in coverage in *Anagrafe* – including irregular and unregistered migrants – currently amounts to approximately 15% of relevant individuals based on the estimates provided by Blangiardo (2016). As a result of this lack of a comprehensive listing, register-based sampling is not recommended in Italy. Istat continues nonetheless to use the *Anagrafe* to conduct immigration surveys, and such surveys are of crucial importance to deepen our knowledge of the foreign population living in Italy and to fill statistical gaps between censuses. However, it is necessary to bear in mind its significant limitations. Most importantly, sampling from the population register leads to selecting only regular and resident migrants, which are the most stable segment of the population of interest (e.g. migrants with a longer length of stay and more frequently reunited with their relatives).

Register-based sampling is on the other hand feasible and adequate in the Spanish case – although its use limits the comparability with sampling efforts in Italy. The remaining challenge in this case is maintaining the costs of surveying a dispersed, relatively mobile and relatively vulnerable population within affordable limits. These problems are compounded by accuracy issues in the register, particularly since (both in the case of Italy and Spain) there is a lack of incentives for immigrants to de-register when they leave the country or change their residence within it. However, two corrective procedures were introduced in Spain in 2007 and 2013, which should have increased the reliability of the registry. In fact, all register-based surveys carried out in Spain following the introduction of these procedures managed to achieve their final numerical targets by applying an exclusively individual random sampling*.*

Still, non-contact rates remain elevated – since immigrants remain a dispersed, relatively hard-to-reach population, and the *Padrón* is still not completely accurate either (Miyar Busto, 2011) despite the significant improvements. So different strategies need to be applied in order to maintain the survey costs within reasonable limits. These methodological solutions include: using the household as a sampling unit; selecting areas of high concentration of the target population; clustering by neighbourhood; or introducing flexibility in contacts replacement and number of trips allowed.

The lack of an exhaustive list of the target population (and the inaccessibility of the *Anagrafe*) has led researchers in Italy to turn to alternative sampling methods which allow including different definitions of migrants (foreign-born) and, in particular, undocumented migrants. In the case of Spain, the financial and administrative barriers to draw and implement samples from the Padrón has also led some researchers to apply alternative sampling methods. The application of these methods faces similar challenges in both countries: ensuring the representativeness and unbiased nature of the sample, and again keeping the costs of sampling under control, due in particular to high attrition rates. In this context, and particularly for the case of Italy, the estimates based on the CS methodology seem to be the most accurate and reliable and the response rates are also satisfactory (Fasani, 2008; Mecatti & Migliorati, 2003; Blangiardo, 2008).

## Footnotes

1 Note that the Italian figures include only foreign citizens, a less encompassing category than foreign-born people. See discussion below.

2 EU-citizens constitute 30% of the foreign residents in Italy (1,5 million) and 32% of the foreign-born residents in Spain (1,9 million).

3 In Italy, both informal processes (in 1986, 1990, 1994, 1998, 2002 and 2006) and formal ones (in 2009 and 2012) have taken place. In Spain, extraordinary regularization processes took place in 1986, 1991/1992, 1996, 2000, 2001, and 2005.

4 Data on foreign-born population is only available in Italy from the decennial census data.

5 EU citizens do not require a residence permit to stay in Italy (or Spain), but they must enroll in the *Anagrafe* (and the Central Registry of Foreigners and Padrón in the case of Spain), when they decide to stay for more than three months. After 5 years, they can apply for the residence card.

6 In addition, a similar number of foreigners were still in the Padrón but deactivated by INE for statistical purposes due to their unclear status (see González-Ferrer, 2009).

7 Since obtaining the permit is not simple or immediate, and this is even more the case when applying (a housing accommodation is mandatory) short-term migrants tend to end up included in irregular or unregistered population.

8 Apart from the ENI2007, INE has not carried out any other survey that focused on the immigrant population but has opted instead by including immigrants as part of the overall sampling design of general surveys, which implies some limitations often related to their small sample sizes.

9 Even this strategy was not sufficient to attain the targeted sample size within the expected calendar, so eventually spatial sampling and random routes were also used to complete the sample (see *Alternative sampling methods for immigrants in Spain and Italy*).

10 Although in principle the official population figures were expected to be cleaned and published in early 2006, the period allowing foreigners affected by the expiry procedure to renew their registration had to be extended and the official cleaned-up figures were not ready until November 2006 (http://www.ine.es/prensa/np447.pdf)

11 MESE stands for the Spanish name of the Project “Migrations between Senegal and Spain”, directed by Pau Baizán, funded by the Spanish Ministry of Science and Innovation (CSO2009-12816). The MESE survey followed the experience of the MAFE Survey in 2008. Detailed information at http://mafeproject.site.ined.fr/en/.

12 Note that this is the longest questionnaire of all surveys with immigrants reviewed in this article (see Table 2). The questionnaire collected information on respondents’ life trajectories in different dimensions (migration, partners, fertility, migration networks, labour, properties, legal status, etc.) year by year, since the date of birth until the date of the interview. The questionnaires are available at http://mafeproject.site.ined.fr/en/.

13 Before discarding an individual, the interviewers had to attempt four visits, one during the weekend and one after 8 pm, in order to maximize the chances of locating the sampled individuals in their homes at some point, and not introducing biases.

14 In MESE, approximately half of the contacts from the *Padrón* list resulted in non-contact, compared to 43-47% in ICS (for Barcelona and Madrid, respectively), 45% in ISSP 2012 and 57% in ISSP 2014.

15 It is highly likely that the good results obtained, despite the specific challenges, are related to some of the specific strategies developed in the implementation of the fieldwork, including: multiple information channels (i.e. letter sent to the domiciles, leaflets, free-call telephone number and email), which helped to build trust, identify specific needs, facilitate contacts and reduce wasted visits; careful selection and training of interviewers, including one Senegalese migrant acting also as mediator; and in-house coordination of the survey for quick evaluation and adjustment of strategies.

16 For a non-comprehensive but illustrative review of different methods developed in the last decades see Reichel and Morales (2017).

17 In Spain, the main sampling method was nonetheless register-based.

18 The non-parametric Kruskal-Wallis and Mann-Withney tests have confirmed that the weight system guarantees the representativeness of the total population by augmenting the representativeness for the most elusive segment of the population (irregular migrants, irregular workers and recently arrived migrants) and solving potential problems of under-coverage for these categories.

19 To identify the centres, a pilot survey using the ‘snow ball sampling’ is recommended, in order to to extend the initial short list into a more exhaustive one, until a sufficiently heterogeneous level is achieved. For full details on the selection procedure and the sampling technique see Baio et al., (2011).

20 As the waves of immigrants of the same nationality follows one another, and the integration process develops, these lists change and the number of centres may decrease, reflecting lower needs for immigrants to remain close to their country fellows. In contrast, when completely new groups arrive, the number of centres grows, including many temporary small spots which are hardly detectable at the early stage of the immigration.
